# Supplementary material for: Mechanisms governing the pioneering and redistribution capabilities of the non-classical pioneer PU.1
Source: Nat Commun. 2020 Jan 21;11:402. doi: 10.1038/s41467-019-13960-2 (PMC6972792; doi:10.1038/s41467-019-13960-2)
Supplement: Supplementary file 7 — Source data [file 41467_2019_13960_MOESM7_ESM.zip › Source_Data/FigureS2/FigureS2B_MotifScanOutput/KmeansCluster10/homerResults/motif17.similar.html]

motif17

## Information for motif17

C
A
T
G
A
T
C
G
G
A
T
C
C
G
T
A
A
C
T
G
A
G
C
T
A
C
G
T
A
G
T
C
G
A
T
C
T
G
A
C
G
C
A
T
A
G
C
T
  
Reverse Opposite:  

C
T
G
A
C
G
T
A
A
C
T
G
C
T
A
G
T
C
A
G
T
G
C
A
T
C
G
A
T
G
A
C
G
C
A
T
C
T
A
G
T
A
G
C
G
T
A
C
  

|  |  |
| --- | --- |
| p-value: | 1e-43 |
| log p-value: | -1.007e+02 |
| Information Content per bp: | 1.695 |
| Number of Target Sequences with motif | 157.0 |
| Percentage of Target Sequences with motif | 4.23% |
| Number of Background Sequences with motif | 428.5 |
| Percentage of Background Sequences with motif | 0.97% |
| Average Position of motif in Targets | 90.8 +/- 53.6bp |
| Average Position of motif in Background | 90.7 +/- 65.7bp |
| Strand Bias (log2 ratio + to - strand density) | -0.8 |
| Multiplicity (# of sites on avg that occur together) | 1.00 |
| Motif File: | file (matrix) reverse opposite |

### Similar de novo motifs found

|  |  |  |  |  |  |  |  |
| --- | --- | --- | --- | --- | --- | --- | --- |
| Rank | Match Score | Redundant Motif | P-value | log P-value | % of Targets | % of Background | Motif file |
| 1 | 0.775 | C G T A C T G A C A G T T C A G C T G A T A C G T G C A T G C A A T G C C G A T A C T G A G T C G A T C | 1e-20 | -47.616005 | 1.29% | 0.18% | motif file (matrix) |
